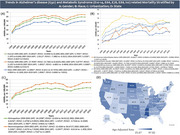# Trends and regional variations of Alzheimer's disease and metabolic syndrome‐related mortality among older American adults from 1999 to 2019

**DOI:** 10.1002/alz70860_101893

**Published:** 2025-12-23

**Authors:** Abdul Moeed, Muhammad Ahmed Ali Fahim, Farah Yasmin

**Affiliations:** ^1^ Dow Medical College, Dow University of Health Sciences, Karachi, Karachi, Sindh, Pakistan; ^2^ Yale School of Medicine, New Haven, CT, USA

## Abstract

**Background:**

Alzheimer's disease poses a significant health burden among older adults in the United States and is highly associated with metabolic syndrome. This study retrospectively analyzes trends and variations in Alzheimer's disease mortality among older adults with metabolic syndrome from 1999‐2019 in the United States.

**Method:**

Our study utilized ICD‐10 codes to analyze death certificate data between 1999 and 2019 from the Centers for Disease Control and Prevention Wide‐Ranging OnLine Data for Epidemiologic Research database for patients aged ≥ 65 years. Age‐adjusted mortality rates (AAMRs), per 100,000 people, and Annual Percentage Change (APCs) and their respective 95% Confidence Intervals (CI) were also calculated for data grouped according to year, gender, race and geography.

**Result:**

Alzheimer's disease in American adults aged ≥ 65 years with metabolic syndrome was responsible for 457,581 deaths. Overall the AAMR increased from 25.14 in 1999 to 39.55 in 2001 (APC: 25.8910*; 95%CI: 13.0083 to 42.849) to 57.3 in 2007 (APC: 5.7935*; 95%CI: 2.1975 to 8.046). A fall in rates to 42.4 by 2014 was observed (APC: ‐2.0112*; 95%CI: ‐7.3802 to ‐0.3521) after which rates continued to increase reaching 58.75 by 2020 (APC: 3.2339*; 95%CI: 0.4637 to 9.8161). Women had higher AAMRs than men (56.51 vs 44.27). The Non‐Hispanic (NH) Black/African American (63) population had the highest AAMRs followed by NH White (52.02), Hispanic/Latino (50.4), NH American Indian/Alaska Native (42.33) and lastly NH Asian/Pacific Islander (34.3). AAMRs were highest in the West (62.33) followed by the South (55.29), Midwest (54.69) and Northeast (34.4). Furthermore, metropolitan areas revealed higher AAMRs (61.63) than nonmetropolitan ones (27.23). States in the top 90th percentile such as Oklahoma, Tennessee, Vermont, North Dakota and Mississippi had over triple the AAMRs when compared with states in the lower 10th percentile including New York, Florida, Nevada, Massachusetts and New Mexico.

**Conclusion:**

Mortality due to Alzheimer's disease in elderly patients with metabolic syndrome has shown a dramatic increase. The highest AAMRs were observed in women, NH Black/African Americans and residents of the West and metropolitan areas. An individualized approach to patient management is necessary moving forward to curb disease progression.